# Performance-Degradation Analysis of the Planetary Roller Screw Mechanism under Multi-Factor Coupling Effects

**DOI:** 10.3390/s24144460

**Published:** 2024-07-10

**Authors:** Kui Chen, Yongsheng Zhao, Jigui Zheng, Wei Shi, Zhaojing Zhang

**Affiliations:** 1 Beijing Key Laboratory of Advanced Ma, Beijing University of Technology, Beijing 100124, China; kchen@emails.bjut.edu.cn; 2 Laboratory of Aerospace Servo Actuation and Transmission, Beijing Research Institute Precision Mechatronics and Controls, Beijing 100076, China; zhengjigui@163.com (J.Z.); njshiwei@163.com (W.S.); jimzzj050@sina.com (Z.Z.)

**Keywords:** planetary roller screw mechanism, performance degradation, transmission accuracy, carrying capacity, efficiency, condition monitoring

## Abstract

The performance-degradation pattern of the planetary roller screw mechanism (PRSM) is difficult to predict and evaluate due to a variety of factors. Load-carrying capacity, transmission accuracy, and efficiency are the main indicators for evaluating the performance of the PRSM. In this paper, a testing device for the comprehensive performance of the PRSM is designed by taking into account the coupling relationships among temperature rise, vibration, speed, and load. First, the functional design and error calibration of the testing device were conducted. Secondly, the PRSM designed in the supported project was taken as the research object to conduct degradation tests on its load-bearing capacity and transmission accuracy and analyze the changes in transmission efficiency. Third, the thread profile and wear condition were scanned and inspected using a universal tool microscope and an optical microscope. Finally, based on the monitoring module of the testing device, the vibration status during the PRSM testing process was collected in real time, laying a foundation for the subsequent assessment of the changes in the performance state of the PRSM. The test results reveal the law of performance degradation of the PRSM under the coupled effects of temperature, vibration, speed, and load.

## 1. Introduction

**Figure 1:**
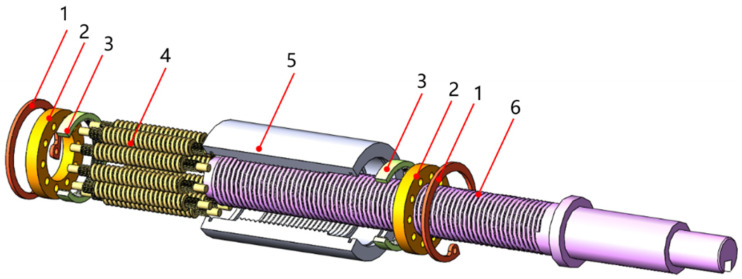
Structure of planetary roller screw mechanism. 1. Elastic retaining ring; 2. cage; 3. internal ring; 4. roller; 5. nut; 6. screw.

As an important actuator element of electromechanical servo actuators with high load-carrying capacity, high accuracy, and high reliability, the planetary roller screw mechanism (PRSM) is widely used in aviation [1], aerospace [2], high-grade CNC machine tools [3], robotics [4,5] and precision electromechanical drives [6,7]. Its structure is shown in Figure 1.

When the PRSM is in motion, the rollers revolve around the screw rod while also revolving around their own axes. Since the rollers and the nut have the same helical rise angle, there is no relative axial displacement between the rollers and the nut during the motion. The helical rise angles of the rollers and the screw are not equal, and there is relative sliding between the rollers and the screw during the motion process, resulting in a large frictional force [8]. Frictional torque is the main factor contributing to variations in the efficiency of PRSM. Load-carrying capacity, efficiency, and transmission accuracy are important indicators for evaluating the transmission performance of the PRSM. Yao et al. [9] established a mathematical model for the load distribution of the PRSM and proposed an improved iterative algorithm to increase the computational efficiency. A multi-objective optimization method was used to obtain the optimal design pitch with the most uniform load distribution at the screw–roller and nut–roller interfaces. Ma et al. [10] established the transmission error calculation models of the PRSM under no-load and different load conditions, respectively. They also conducted experimental verification. Du Xing et al. [11,12] established a mechanical model containing radial load and machining error, and the results proved that both load distribution and fatigue life change significantly with the change of machining errors. Qiao et al. [13] used the finite-element method to simulate and analyze the steady-state thermal and transient thermal force coupling of a planetary roller screw mechanism. The results proved that the rotational speed and external load have a great influence on the thermal characteristics of the PRSM. It provides a basis for the study of the effect of thermal errors on the performance of the PRSM. Niu et al. [14] proposed a fault diagnosis method for the PRSM that combines a seeded bird-flocking algorithm with a support vector machine. This method extracts vibration eigenvalues from the time domain, frequency domain, and time–frequency domain, respectively, which is significant for monitoring the health status of the PRSM during operation. Yang et al. [15,16] proposed an adaptive surrogate-assisted multi-objective evolutionary algorithm decomposition framework, which solved the problem of multi-objective optimization and provided a useful reference for exploring the laws of performance degradation of the PRSM under the coupling effect of multiple factors. Meng et al. [17] established a multiscale adhesive wear model for the PRSM and analyzed the influence law of wear on the transmission accuracy of the PRSM. Zhang et al. [18] introduced an enhanced method for optimizing load distribution in the PRSM. This method involves modifying the mesh contact conditions between the rollers, screw, and nut, ensuring that loads are evenly distributed across each thread of the PRSM. Xie et al. [19] established a hybrid lubrication model for the PRSM and analyzed the effects of screw speed, axial load, and surface roughness on lubrication performance. Improving machining accuracy to reduce surface roughness can improve the lubrication condition and reduce friction and wear. Zhang et al. [20] developed an analytical model of the electromechanical servo system of the PRSM, and the results proved that, when the PRSM is subjected to friction, the response of the system slows down. The larger the gap or the smaller the stiffness, the larger the wave amplitude of the system response. Abevi et al. [21] investigated the static mechanical properties of the PRSM and obtained the global and local deformations in different configurations by experimental and finite-element methods. Ma et al. [22] consider the effect of the mounting method of the PRSM and use three-dimensional finite-element analysis to simulate and evaluate the load-distribution characteristics under thermo-mechanical coupling. Du et al. [23] developed a simplified kinetic model in order to study the thermal characteristics of the PRSM. The temperature-field distribution of the PRSM was analyzed using the finite-element method and experimentally verified. Li et al. [24] designed a high-load, high-performance PRSM and analyzed its load-carrying performance and transmission efficiency using the finite-element method.

Most of the above studies on the performance of the PRSM have focused on theoretical studies, and most of the theoretical studies have carried out idealized assumptions in terms of condition setting, which has led to a certain bias in the prediction and evaluation of the performance of the PRSM. In this paper, the influence law of various factors on the performance degradation of the PRSM is analyzed from the perspective of experimental testing. To this end, a comprehensive performance-testing device for the PRSM was designed, and degradation tests were conducted on the load-bearing capacity and transmission accuracy. Quantitative analysis was carried out on the influence of various factors on the performance degradation of the PRSM, providing technical support for the performance testing of the PRSM. The tooth shape and wear condition of the thread were scanned and inspected using a universal tool microscope and an optical microscope, revealing the mechanism of performance degradation of the PRSM. At the same time, the vibration conditions of the PRSM under different performance states were collected and statistically analyzed, and a preliminary exploration was conducted on the characterization features for the performance state assessment of the PRSM.

## 2. Development of a Comprehensive Performance Test Device for the PRSM

To thoroughly investigate the intricate mechanisms underlying the performance degradation of the PRSM, which are influenced by the complex interaction of temperature rise, vibration, speed, and load, a loading test device and a transmission-accuracy test device for the PRSM were designed. Loading tests and transmission-accuracy tests of the PRSM under different working conditions can be carried out. It provides valuable data support for elucidating the mechanism of performance degradation in the PRSM.

### 2.1. Development of the PRSM Loading Test Device

**Figure 2:**
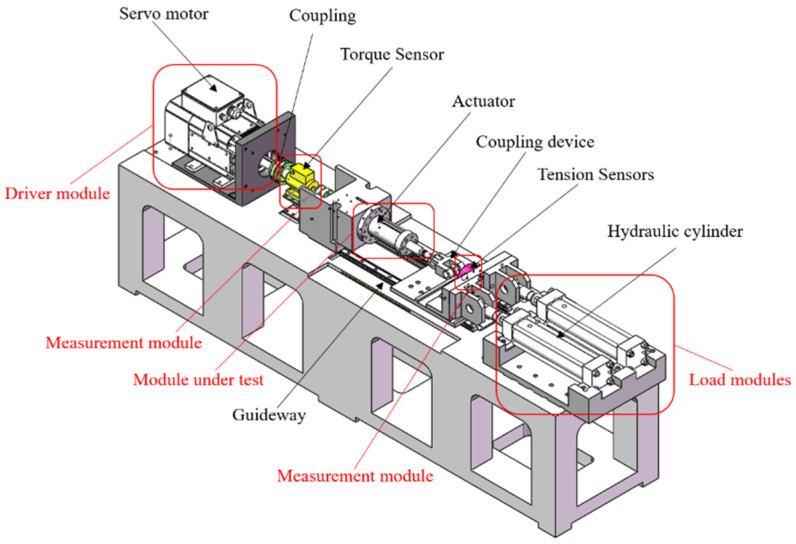
Design scheme for the loading test device of the PRSM.

The structure of the loading performance test device is shown in Figure 2. It includes a motor drive module, torque speed sensor, angle grating sensor, tensile pressure sensor, temperature sensor, vibration sensor, axial negative-loading module, data-acquisition module, and measurement and control software, among other parts. It can be used to measure static and dynamic load tests on the PRSM. Data such as torque, speed, temperature rise, vibration, and tensile strength can be recorded and stored in real time during the test.

The hydraulic system serves as a passive loading source to provide axial loads, and it can achieve the loading, holding, and unloading of loads by setting the loading rate. The range of the axial loads is 0–100 kN. To eliminate the influence of eccentric loading, dual hydraulic cylinders are used for loading, and displacement sensors are employed to achieve synchronous control of the dual hydraulic cylinders.

Due to the characteristics of the tensile and compressive sensor, an open loop is formed between the loading chain and the driving chain at this point, resulting in coaxiality errors between the loading chain and the driving chain. This will cause the tested PRSM to be affected by the overturning moment, leading to premature damage, failure of the tested PRSM, and inability to obtain accurate test data. To compensate for the coaxiality errors between the loading chain and the driving chain, a double-ended hinge connection method is designed to reduce the impact of the overturning moment.

### 2.2. Development of Test Equipment for the PRSM

**Figure 3:**
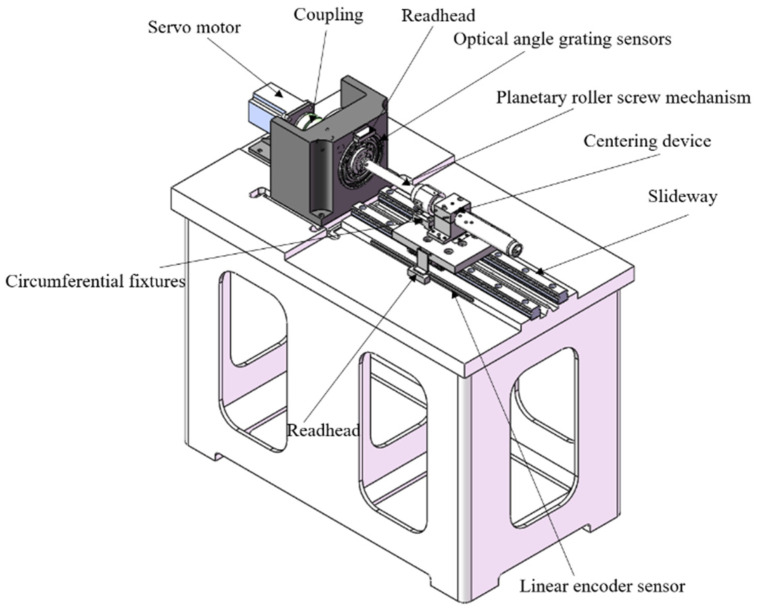
Design scheme of precision measuring device for the PRSM.

In order to test the degradation trend of the transmission accuracy of the PRSM, a transmission-accuracy testing device was developed. Its measurement principle is to convert the rotation angle value of the PRSM collected by the circular grating sensor into a linear displacement value and, then, compare it with the linear displacement value of the PRSM collected by the linear grating sensor, thereby obtaining the position deviation of the data-collection point during the PRSM testing process. Its structural design is shown in Figure 3.

The precision testing device for the PRSM transmission includes a servo motor drive module, a circular grating sensor, a linear grating sensor, a data-acquisition module, and a support module. It can achieve real-time collection, processing, and visual display of test data, and store the data in a designated path. The device is equipped with testing functions for pitch error, lead error, positioning error, and repeated positioning error.

### 2.3. Consistency Verification of the Test Device

Consistency verification of the developed testing device is an important step to examine whether the testing device can truly reflect the actual performance of the PRSM under test. It is also an important indicator for verifying the rationality of the design of the testing device.

#### 2.3.1. Consistency Verification of the PRSM Loading Test Device

**Figure 4:**
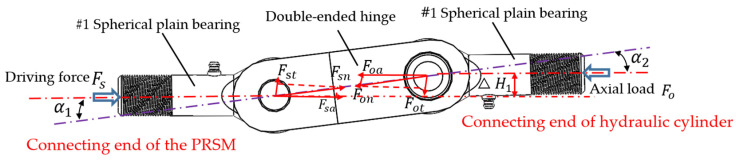
Structure and stress analysis of double-ended hinge.

Due to the structural characteristics of the tension–compression sensor, there is a coaxiality error between the drive chain and the loading chain after the load is applied, which leads to premature failure and damage to the PRSM. The drive chain refers to the motion transmission chain composed of a servo motor, a coupling, and a tested PRSM. The loading chain refers to the load transmission chain composed of a hydraulic cylinder, a force transmission module, and a tension–compression sensor. To solve this problem, a double-ended hinge connection structure was designed, as shown in Figure 4. According to the structural and stress analysis of the double-ended hinge, its connecting structure can compensate for the coaxiality error between the drive chain and the loading chain, thus improving the accuracy of loading tests.

To verify the consistency of the performance of the loading test device, 15 sets of loading tests were conducted on two PRSM products of the same batch and specifications, respectively. In each set of loading tests, the PRSM operated reciprocally three times. The driving torque of the servo motor was collected and processed during the testing process. The changes in the driving torque under the same working conditions were compared to verify the consistency of the loading test device.

The PRSM is lubricated with grease, and the setup conditions for the loading test are shown in Table 1.

**Figure 5:**
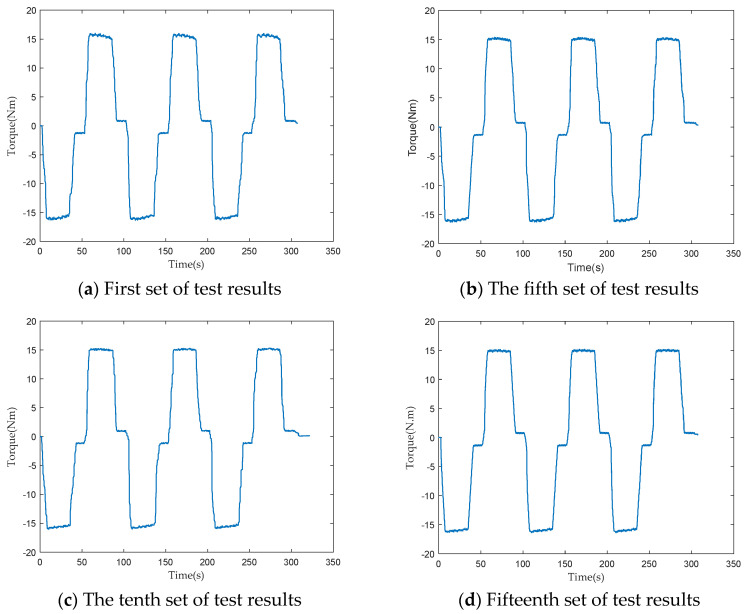
Torque test results of the first PRSM loading experiment.

**Figure 6:**
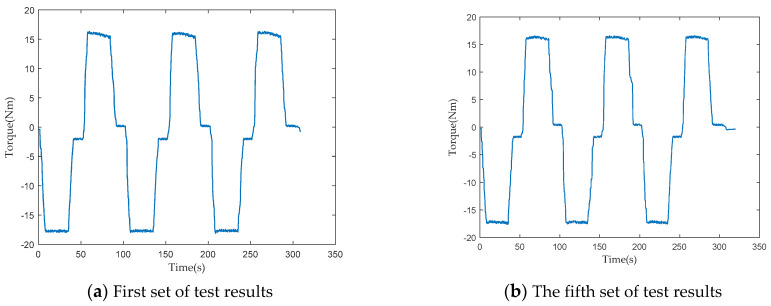

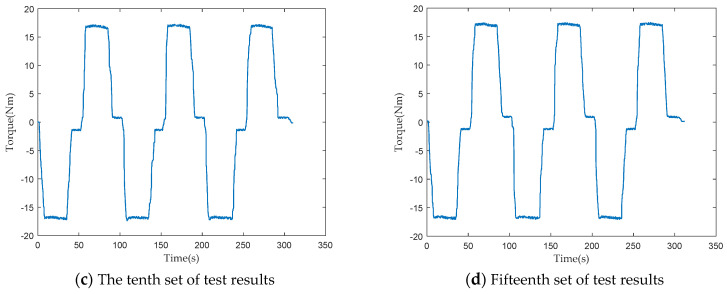
Torque test results of the second PRSM.

The loading test results of the two PRSMs are shown in Figure 5 and Figure 6. According to the test results, under the same axial load, the driving torques of the two PRSMs during the forward and reverse stroke tests do not differ much and have the same trend of change. This difference is caused by the precision of the processing and assembly of the PRSMs. It shows that the loading test device has good consistency and can reflect the performance differences between different PRSMs.

#### 2.3.2. Consistency Verification of the Accuracy Test Device

**Figure 7:**
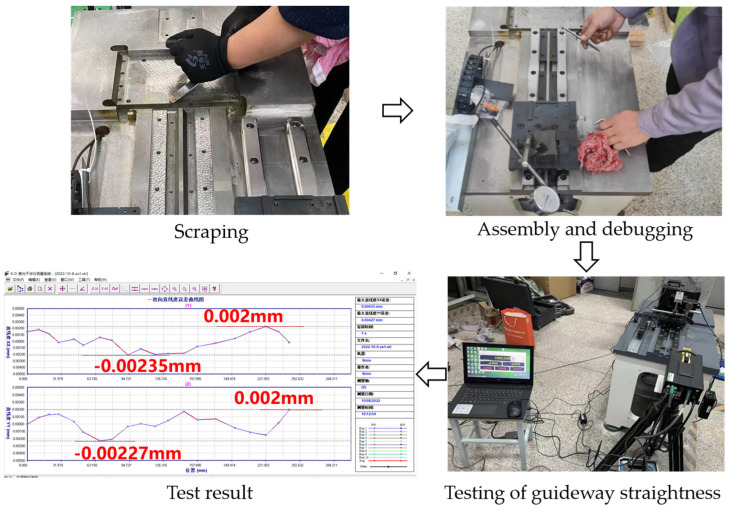
Assembly debugging and straightness test of the guide rail.

To improve the test precision of the transmission precision test device, the installation datum plane of the guide rail has undergone scraping treatment. After the completion of the assembly and debugging of the guide rail, a laser interferometer is used to test the straightness of the assembled guide rail. The test results are shown in Figure 7. According to the results, the yaw error after the completion of the guide-rail assembly is 0.00435 mm, and the pitch error is 0.00427 mm.

To verify the consistency of the transmission-accuracy test device, the initial transmission accuracy of two PRSMs of the same batch and specifications was tested separately. The initial transmission-accuracy test results of the two PRSMs were compared and analyzed. The setting of the test conditions for the transmission accuracy of PRSM is shown in Table 2.

**Figure 8:**
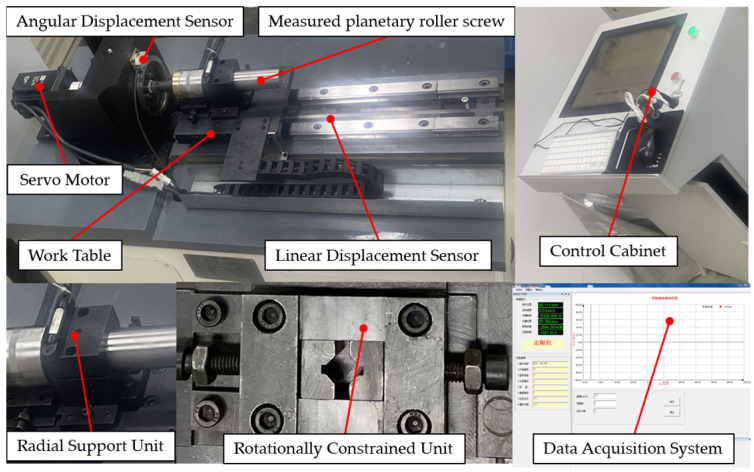
The testing process of the initial transmission accuracy of the PRSM.

**Figure 9:**
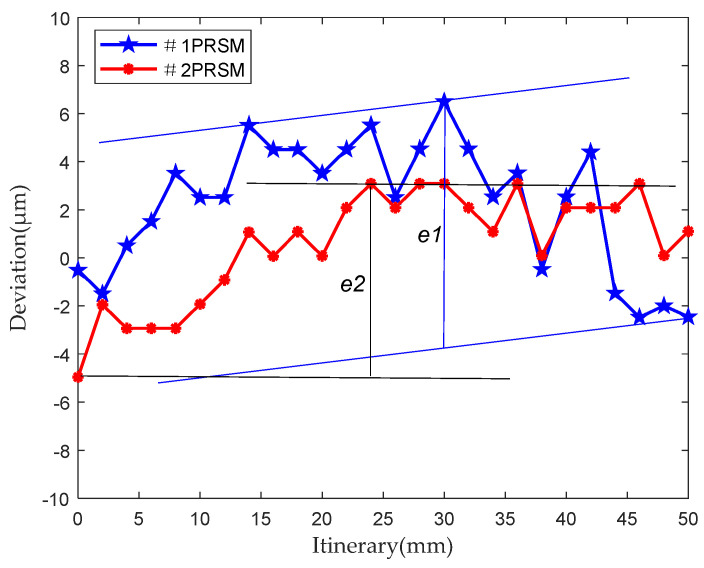
The results of the initial transmission-accuracy test for the PRSMs.

The initial transmission accuracy of two PRSMs produced in the same batch, numbered 1 and 2, respectively, is tested. The test conditions are set as shown in Table 2. The testing process of the transmission accuracy of the PRSM is shown in Figure 8. The test results of the initial transmission accuracy of the PRSMs are shown in Figure 9.

According to the test results, the initial transmission accuracy of #1PRSM is 10.254 μm, while the initial transmission accuracy of #2PRSM is 8.044 μm. Moreover, the position deviation curves of both of them show similar trends, indicating that the transmission-accuracy test device can not only measure the transmission accuracy of the PRSM but also reflect the differences between them. The difference in transmission accuracy between the two may be caused by processing errors and assembly errors.

## 3. Performance-Degradation Experiment of the PRSM

### 3.1. Transmission-Accuracy Test of the PRSM

**Figure 10:**
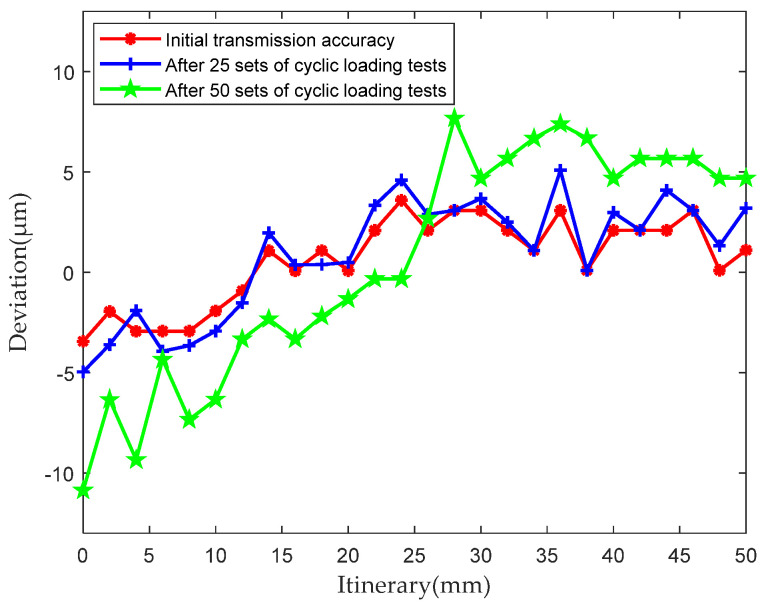
Measurement results of positioning error of the PRSM.

The steps for testing the transmission-accuracy degradation of the PRSM are as follows. (1) Conduct the initial transmission-accuracy test of the PRSM. (2) Conduct the transmission-accuracy test after 50 min of load test. (3) Conduct the transmission-accuracy test after 100 min of load test. The test results of the transmission-accuracy degradation of #3 PRSM are shown in Figure 10.

The results indicate that, as the loading test proceeds, the transmission accuracy of the PRSM shows a trend of degradation. The initial transmission accuracy is 7.024 μm. After 50 min of loading test, the transmission accuracy becomes 9.127 μm, with a degradation of 29.9%. After 100 min of loading test, the transmission accuracy degrades to 17.65 μm, with a degradation of 93.4%. At this point, although the threads of the PRSM have not yet been damaged or failed, its transmission accuracy can be considered as completely degraded. The results demonstrate that, with the decrease in the load-bearing capacity of the PRSM, its transmission accuracy exhibits an accelerated degradation trend. 

### 3.2. Load-Bearing Performance Tests of the PRSM

#### 3.2.1. Loading Test Conditions for the PRSM

The structural parameters of the PRSM designed in this test are shown in Table 3. Its rated load is 20 kN. To shorten the testing time and obtain the degradation trends of bearing performance and transmission accuracy, an axial load of 30 kN is used for the loading test.

The loading test process of the PRSM is as follows. (1) The PRSM to be tested is installed on the loading test device. (2) Open the testing software and set the data-saving path. (3) Set the stroke and number of cycles for the loading test. (4) Set the acquisition frequency for the torque signal, temperature-rise signal, and vibration signal. (5) Set the axial load, as well as the loading rate and unloading rate of the load. (6) Set the load control function module to maintain 0 kN. (7) Click the test button to start the loading test. 

**Figure 11:**
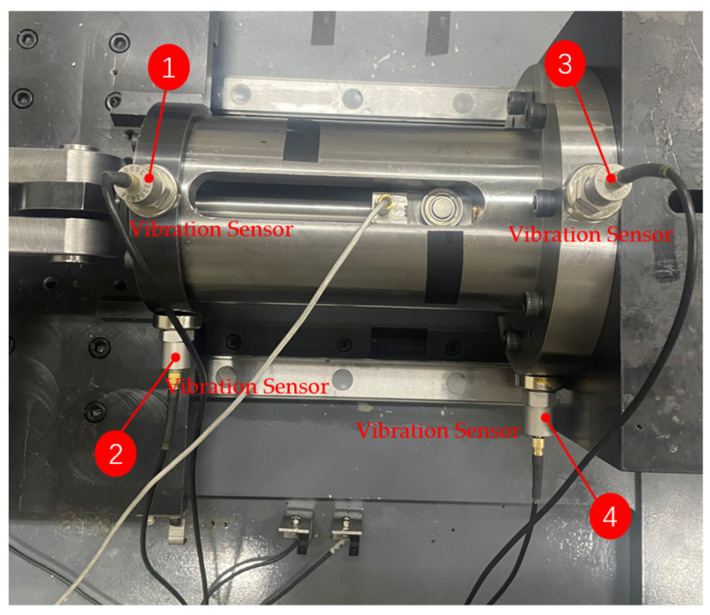
Arrangement of vibration sensors.

**Figure 12:**
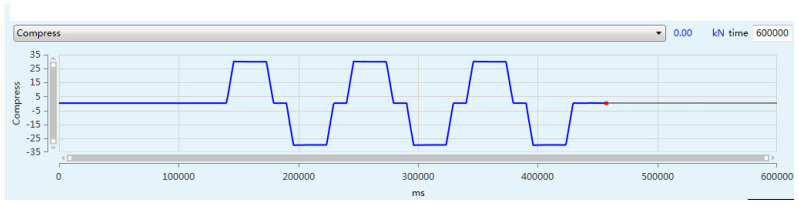
Axial load spectrum.

**Figure 13:**
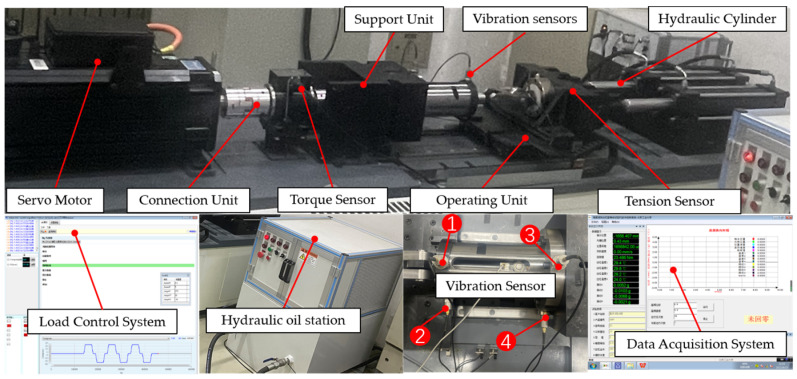
Loading test process of the PRSM.

To analyze the changing trend of the vibration state of the PRSM as its performance degrades, the vibration state of the PRSM is collected in real time during the loading test. The vibration sensors are arranged on the outer side of the actuator tooling of the PRSM, as shown in Figure 11. Vibration sensors #1 and #2 are placed at the front end of the actuator cylinder, which is far away from the motor. Vibration sensor #1 is placed vertically, with upward as the positive direction. Vibration sensor #2 is placed horizontally, with leftward as the positive direction. Vibration sensors #3 and #4 are placed at the rear end of the actuator cylinder, which is close to the motor. Vibration sensor #3 is placed vertically, with upward as the positive direction. Vibration sensor #4 is placed horizontally, with leftward as the positive direction. The temperature sensor is arranged on the outer circular surface of the nut to monitor the temperature change of the PRSM during the loading test. The conditions set for the loading test of the PRSM are shown in Table 4. The setting of the axial load spectrum is shown in Figure 12. At the start of the test, a small load is first applied to eliminate the engagement clearance between the threads. After that, the load is increased to the set value at a certain rate. Similarly, during reverse operation, a small load is first applied to eliminate the engagement clearance between the threads. After that, the load is increased to the set value at a certain rate. The cyclic loading test process of the PRSM is shown in Figure 13.

#### 3.2.2. Load-Bearing Performance-Degradation Test of the PRSM 

**Figure 14:**
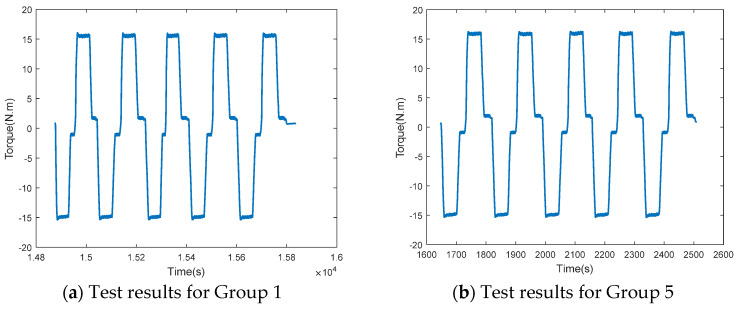

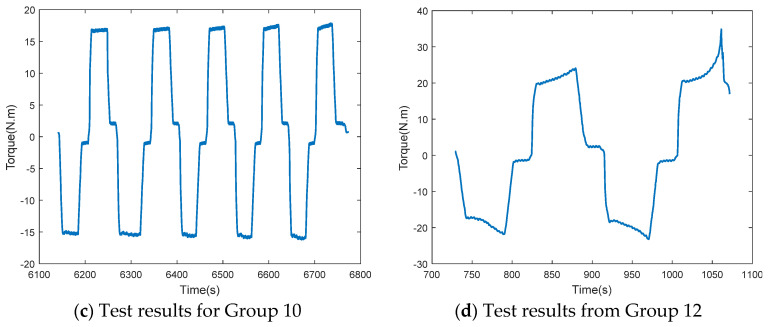
The test results of the degradation in bearing performance of the PRSM.

**Figure 15:**
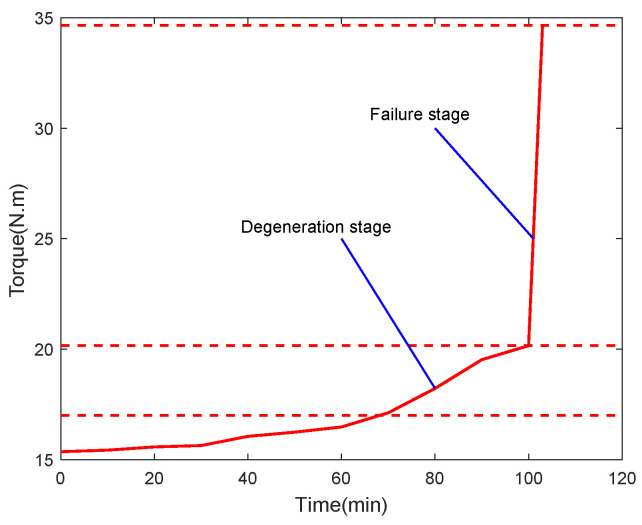
Variation trend of driving torque of the PRSM.

The results of the loading test for the PRSM are shown in Figure 14. According to the test results, during the first five sets of loading tests, the driving torque of the PRSM remained stable at around 15.5 N⋅m for the forward stroke and around −15 N⋅m for the reverse stroke, with no significant changes. However, during the loading tests from group 5 to group 10, the driving torque increased from 15.5 N⋅m to 18.4 N⋅m for the forward stroke, and from −15.3 N⋅m to −17.6 N⋅m for the reverse stroke. During this phase, the bearing performance of the PRSM began to show signs of degradation. During the loading tests from group 10 to group 11, the driving torque of the forward stroke increased from 18.4 N⋅m to 20.2 N⋅m, while the driving torque of the reverse stroke increased from −7.5 N⋅m to −19.8 N.m. During this phase, the bearing performance of the PRSM exhibited an accelerated trend of degradation. During the 12th set of loading tests, the driving torque of the reverse stroke increased from −20.2 N⋅m to −23.6 N⋅m, and the driving torque of the forward stroke suddenly increased from 24.2 N⋅m to 35.4 N⋅m, indicating that the PRSM had suffered damage and failed. The changing trend of the driving torque during the forward stroke test of the PRSM is shown in Figure 15.

### 3.3. Transmission-Efficiency Calculation of the PRSM

**Figure 16:**
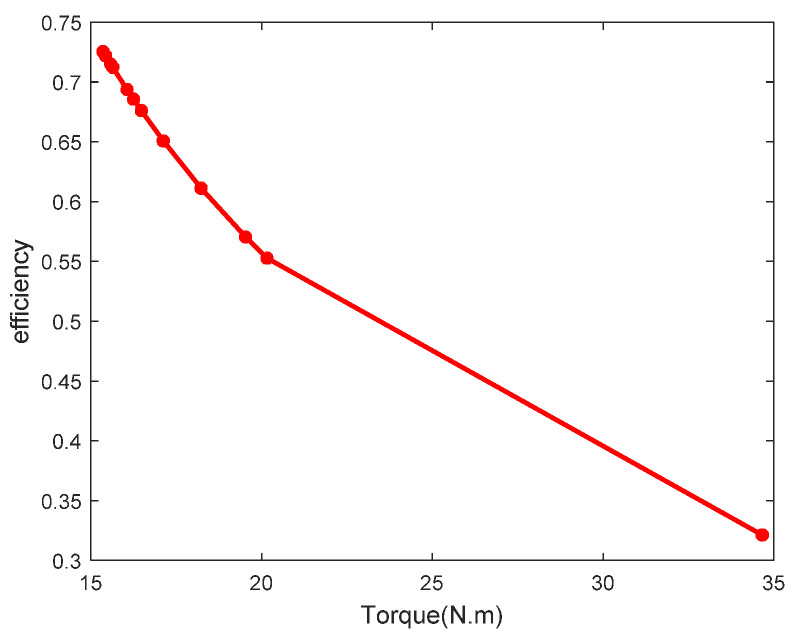
Changing trend of transmission efficiency of PRSM.

Transmission efficiency is an important indicator for evaluating the performance of the PRSM. According to the literature [8], the test data are processed to analyze the changing trend of the transmission efficiency of the PRSM, and the results are shown in Figure 16. According to the results, under the axial load of 30 kN, when the driving torque of the PRSM increases from 15 N⋅m to 17 N⋅m, the transmission efficiency of the PRSM decreases slightly. When the driving torque of the PRSM increases from 17 N⋅m to 23 N⋅m, the transmission efficiency rapidly decreases. When the driving torque suddenly increased to 35.4 N⋅m, the PRSM failed.

**Figure 17:**
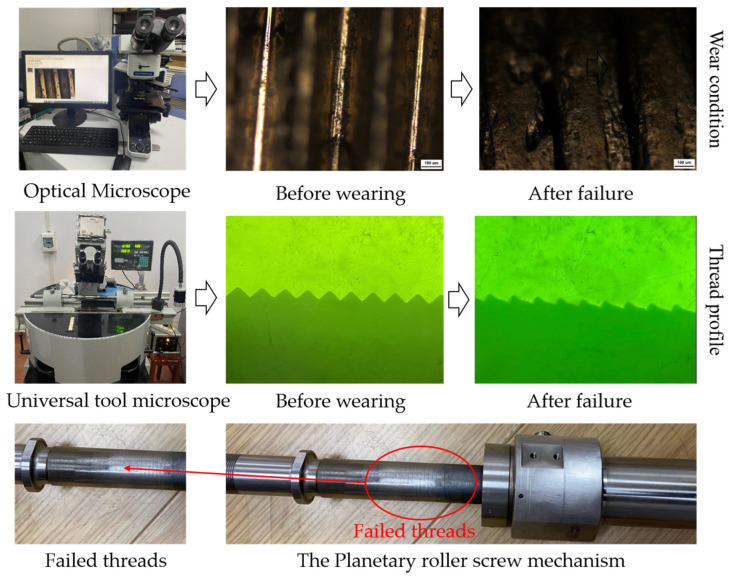
Wear condition of screw threads.

**Figure 18:**
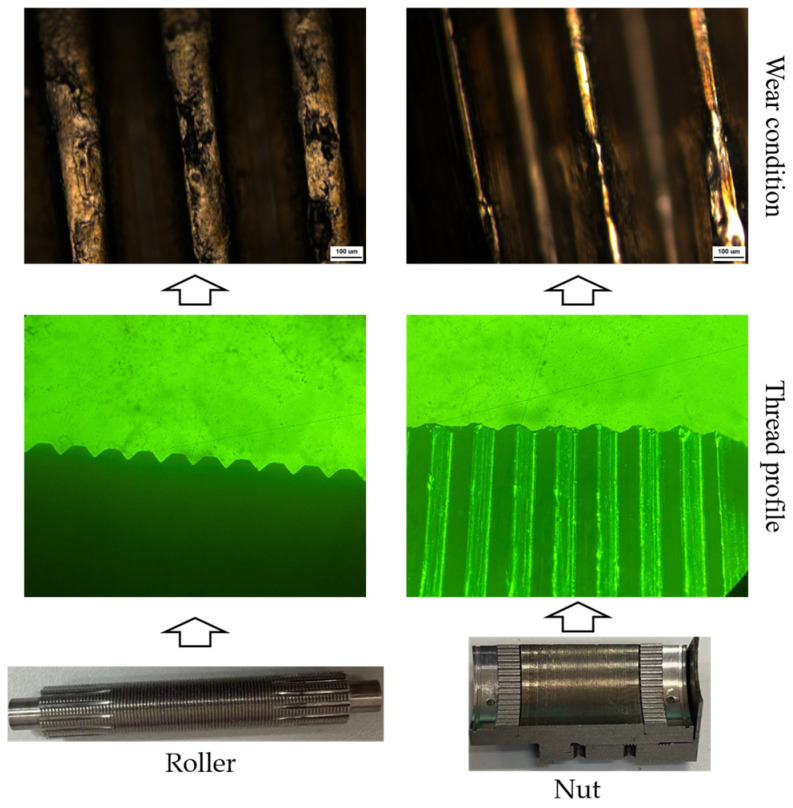
Wear condition of roller threads and nut threads.

The deformation and wear state of the screw rod threads were inspected using a universal tool microscope and an optical microscope, and the results are presented in Figure 17. The findings reveal severe deformation of the screw threads, accompanied by wear and material spalling on the threads. The wear condition of the roller thread and nut thread is shown in Figure 18. According to the results, deformation and wear have occurred on the roller threads and nut threads. The wear degree of the roller thread is higher than that of the nut thread but lower than that of the screw thread. This suggests that material spalling is the primary factor contributing to the degradation of the load-bearing performance and transmission accuracy of the PRSM.

## 4. Operating Condition Monitoring Analysis of the PRSM

### 4.1. Temperature-Rise Monitoring of the Nut

**Figure 19:**
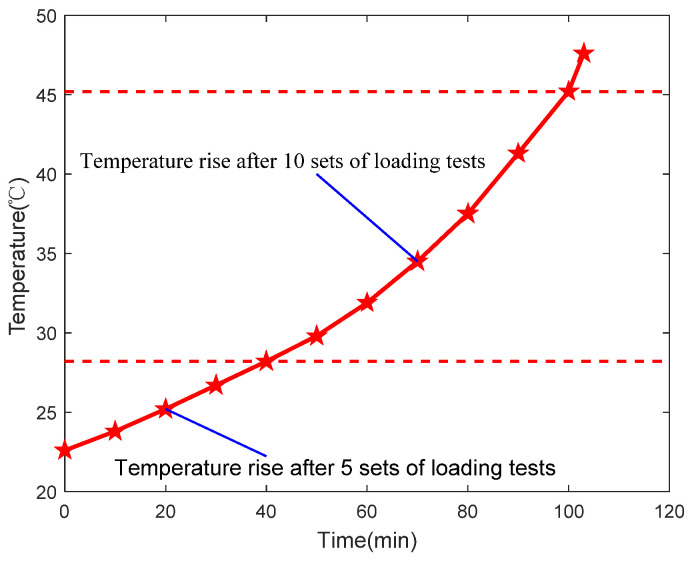
Temperature trend at the outer circle of the nut.

A temperature sensor is installed at the step of the outer circle of the nut to monitor the temperature rise during the loading test, and the temperature-rise detection results are shown in Figure 19. According to the test results, the temperature of the outer circle of the nut rose by 5.6 °C after five sets of loading tests. After twelve sets of cyclic loading tests, the temperature increased by 19.4 °C. During the first five sets of loading tests, the temperature rise of the cylindrical surface of the nut was gradual. However, from the fifth set to the twelfth set of loading tests, the rate of temperature rise on the outer circular surface of the nut accelerated. This indicates that, as the loading test progressed, the wear between the engaged threads worsened, leading to increased friction heat generation and a degradation of the performance of the PRSM.

### 4.2. Vibration Condition Monitoring

**Figure 20:**
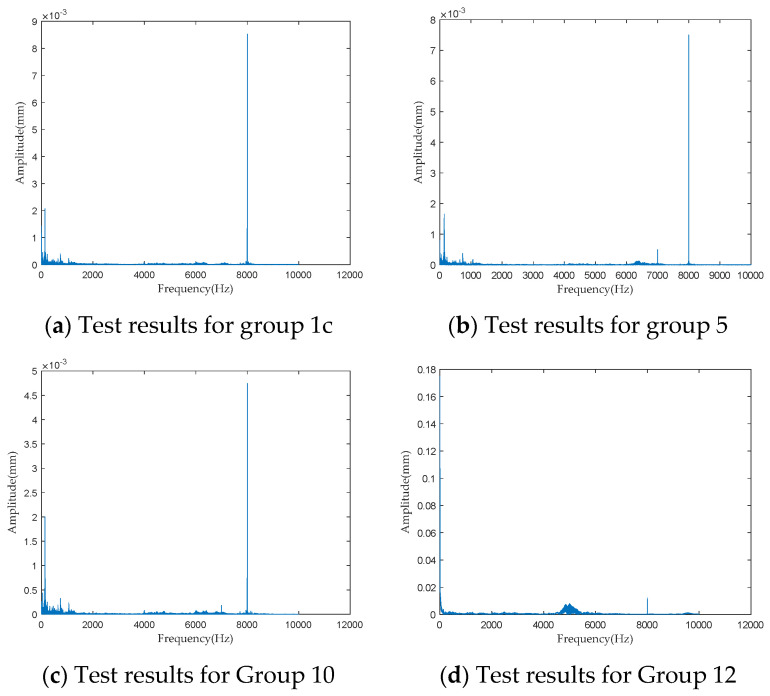
Test results of vibration sensor #1.

**Figure 21:**
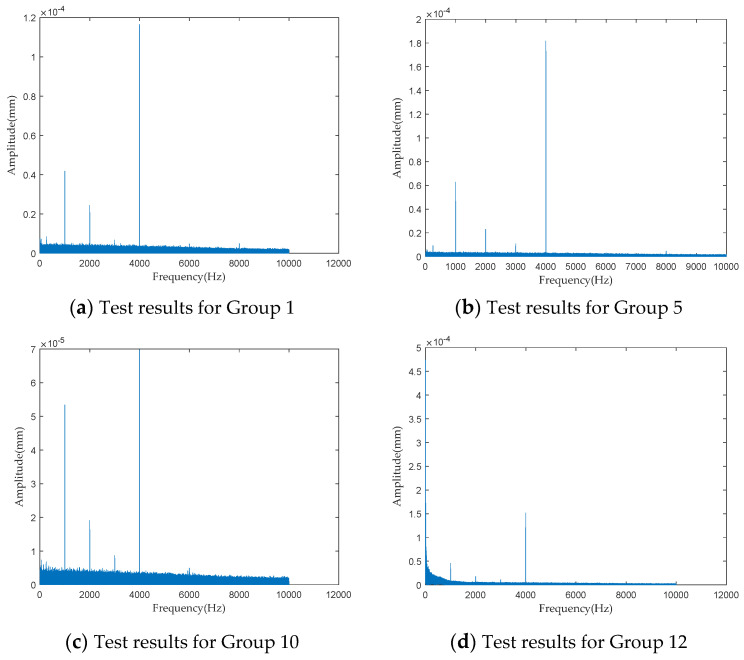
Test results of vibration sensor #2.

**Figure 22:**
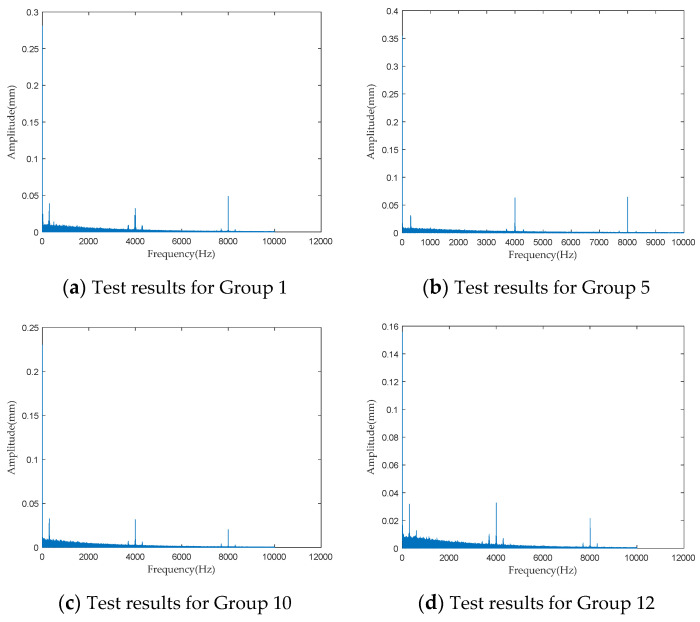
Test results of vibration sensor #3.

**Figure 23:**
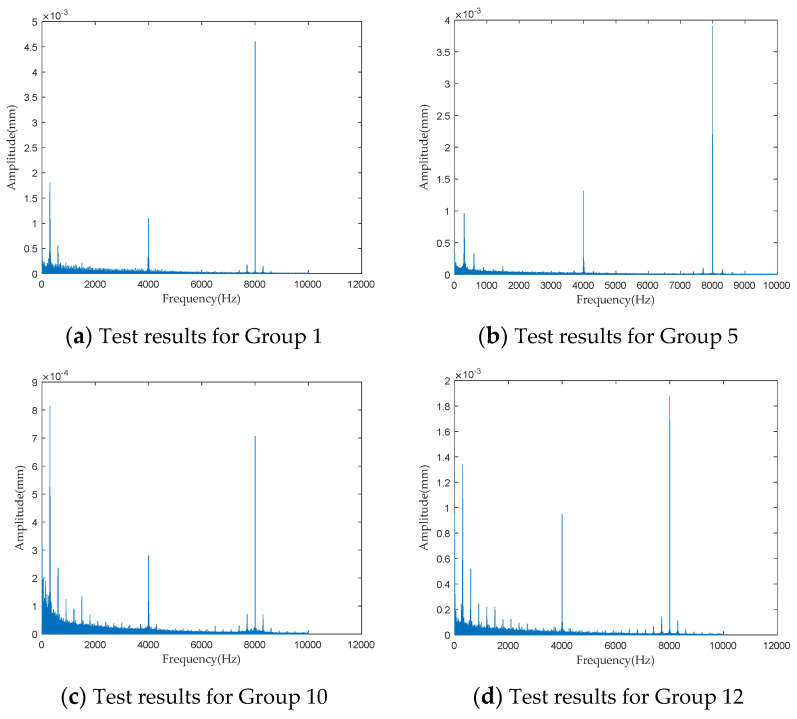
Test results of vibration sensor #4.

**Figure 24:**
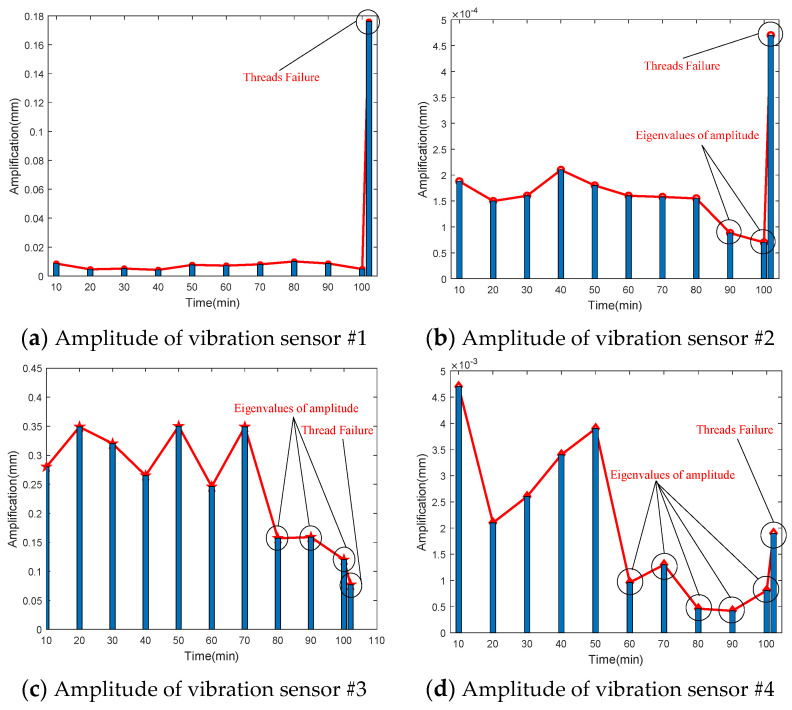
Trend of maximum magnitude of vibration sensors.

To investigate the impact of performance degradation on the vibration state of the PRSM, four vibration sensors were arranged on the outer cylindrical surface of the actuator cylinder. The vibration data were processed using MATLAB R2018b, and the results are shown in Figure 20, Figure 21, Figure 22 and Figure 23. Taking the amplitude of the frequency domain signal as the research object, the influence of planetary degradation on the amplitude is analyzed. According to the processing results of the data collected by vibration sensor #1, it can be seen that the amplitude value reached the maximum when the PRSM failed. Based on the processing results of the data collected by vibration sensor #2, it can be seen that, with the degradation of the performance of the PRSM, its amplitude value showed a trend of first decreasing and then increasing, reaching the maximum when the PRSM failed. Based on the processing results of the data collected by vibration sensor #3, it can be seen that, as the performance of the PRSM degrades, its amplitude value exhibits a decreasing trend and reaches its minimum when the PRSM fails. Based on the processing results of the data collected by vibration sensor #4, it can be seen that, with the degradation of the performance of the PRSM, its amplitude value first decreases and then increases, but it does not reach the maximum value when the PRSM fails. The trend of vibration amplitude changes during the loading test of the PRSM is shown in Figure 24. According to the test results, the vibration state of the PRSM changes as its performance degrades. However, the trends at different locations are not consistent. When using vibration signals as a basis to determine the performance status of the PRSM, it is necessary to study the placement method and location of the vibration sensors to improve the accuracy of the assessment.

During the load test, the variance changes of the vibration signal are shown in Table 5. From the results, it can be seen that the changing trends of the variance values of the vibration signals at different locations are not consistent. The variance values of the vibration signals at Position 1 and Position 2 show an increasing trend as the loading test progresses. The variance values of the vibration signals at Position 3 and Position 4 show a decreasing trend as the loading test progresses.

**Figure 25:**
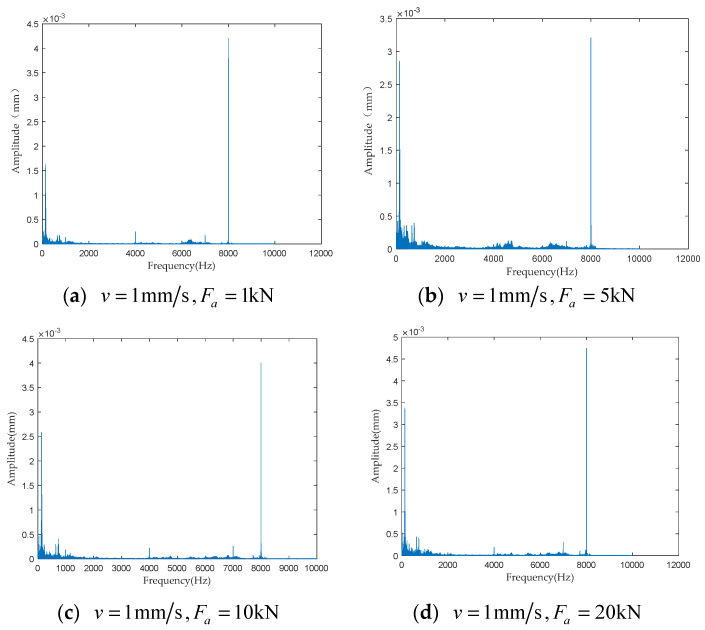
Test results of vibration sensor #1.

**Figure 26:**
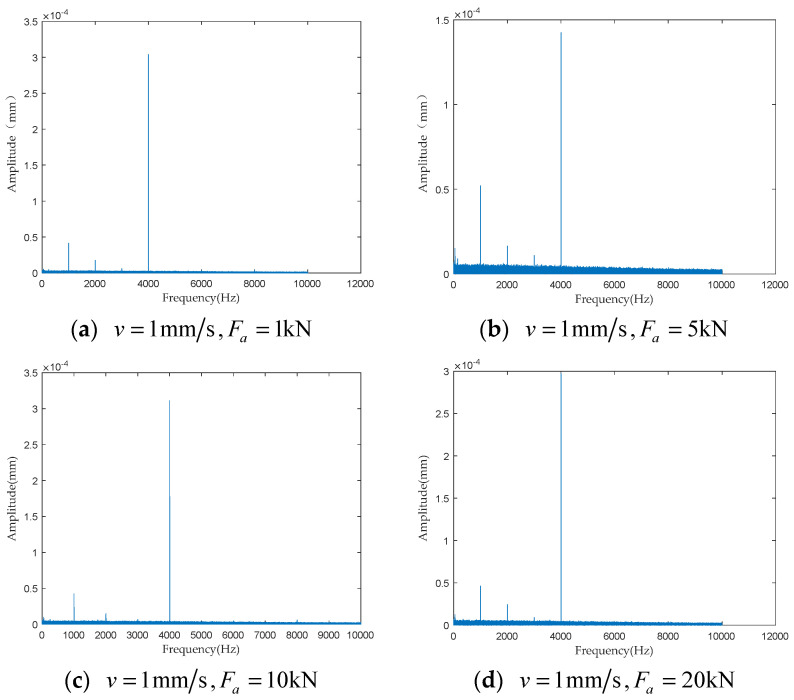
Test results of vibration sensor #2.

**Figure 27:**
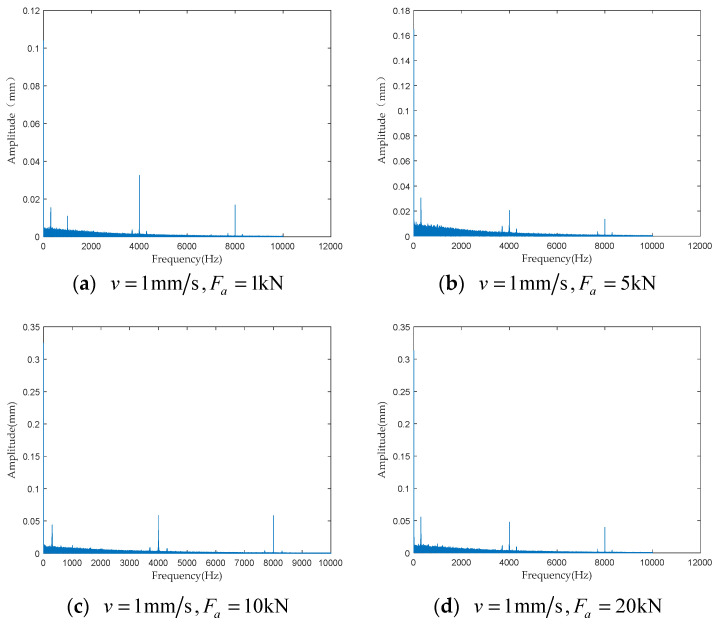
Test results of vibration sensor #3.

**Figure 28:**
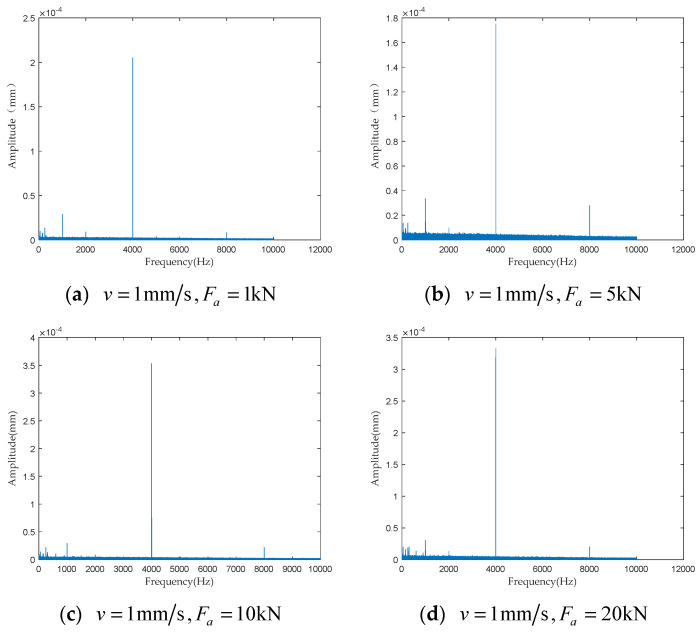
Test results of vibration sensor #4.

**Figure 29:**
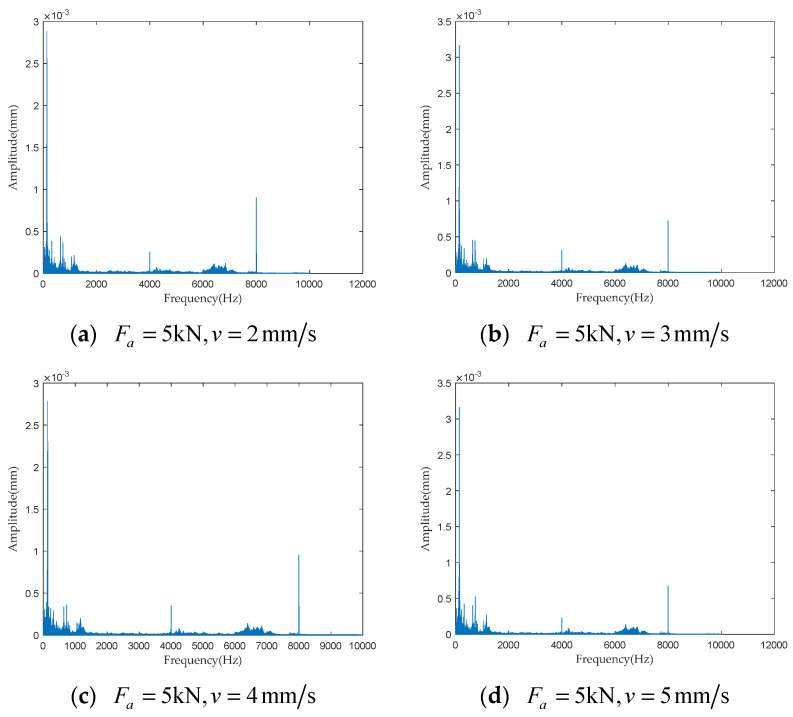
Test results of vibration sensor #1.

**Figure 30:**
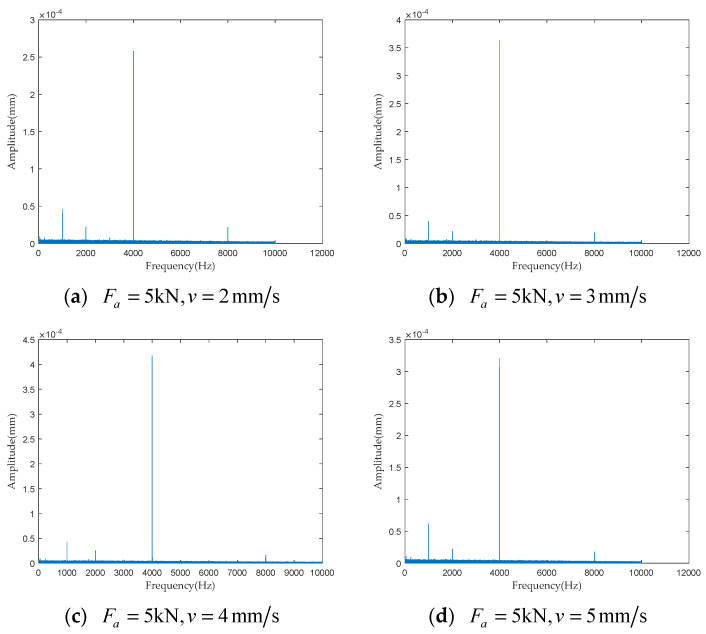
Test results of vibration sensor #2.

**Figure 31:**
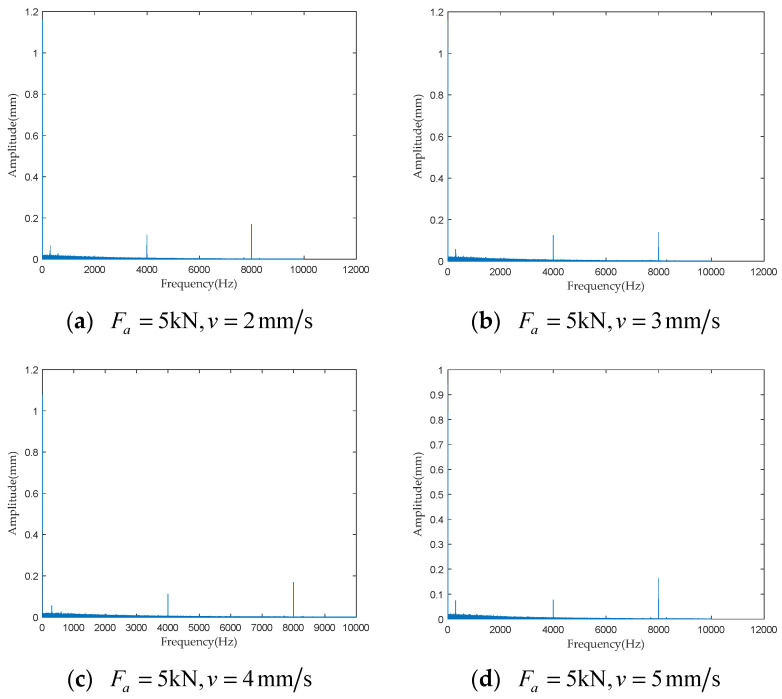
Test results of vibration sensor #3.

**Figure 32:**
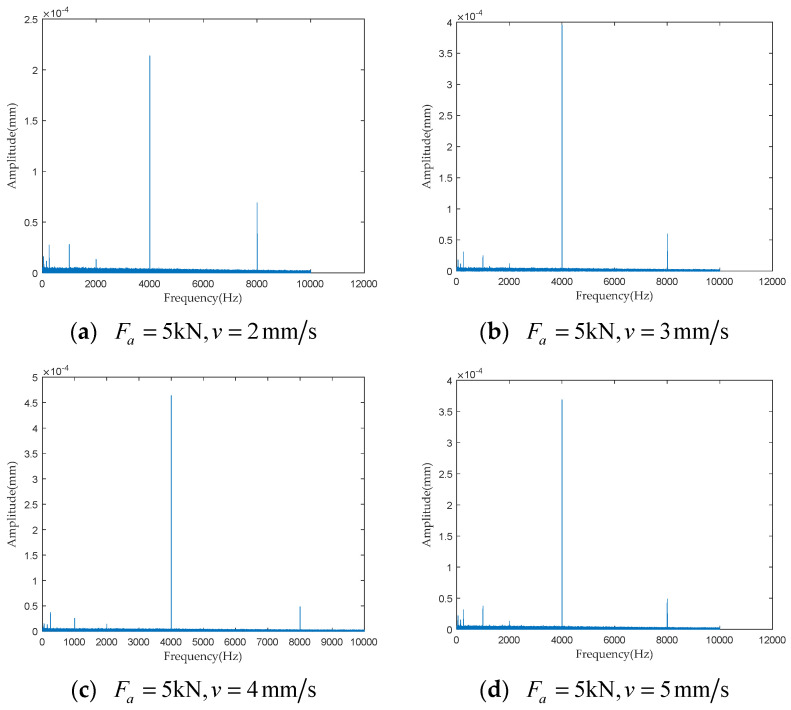
Test results of vibration sensor #4.

To further investigate the impact of performance degradation of the PRSM on its vibration state, loading tests were conducted at different speeds and axial loads to detect the vibration state of the PRSM. When the running speed of the PRSM was constant, the axial loads were set to 1 kN, 5 kN, 10 kN, and 20 kN, respectively. The vibration state calculation results are shown in Figure 25, Figure 26, Figure 27 and Figure 28. From the test results, it can be observed that, when the running speed of the PRSM is constant and only the axial load is changed, the vibration amplitude of the PRSM does not undergo significant variations. When the axial load on the PRSM is unchanged, the speeds of the PRSM were set to 2 mm/s, v=3 mm/s, v=4 mm/s, v=5 mm/s, respectively. The vibration state calculation results are shown in Figure 29, Figure 30, Figure 31 and Figure 32. According to the results, when the axial load of the PRSM remains constant and the speed of motion is changed, the vibration amplitude of the PRSM does not undergo significant changes. This proves that, when the performance of the PRSM has not deteriorated, its vibration state will not change due to variations in axial load and speed. This indicates that changes in the vibration state of the PRSM can be used to characterize the state of performance degradation.

## 5. Conclusions

Performance testing is an important step to verify the performance of the PRSM and is an essential means for confirming the maturity level of the product. This paper has developed a comprehensive performance-testing device for the PRSM and verified its consistency. The load-bearing performance and transmission-accuracy degradation of the PRSM were tested, and the following conclusions were drawn.

1. The degradation of the load-bearing performance of the PRSM can be divided into three stages: the normal degradation stage, the accelerated degradation stage, and the failure stage. The rate of degradation of load-bearing performance gradually increases;
2. Under the axial load of 30 kN, the screw threads failed first. The deformation and wear of the threads are the main factors causing the performance degradation of the PRSM;
3. When the performance of the PRSM is not degraded, its vibration state is unrelated to the operating speed and axial load. However, when the performance of the PRSM starts to degrade, its vibration state will change accordingly, and the performance status of the PRSM can be evaluated based on the changes in the vibration state. Nevertheless, the vibration state trends vary at different locations, necessitating further research on the placement and manner of vibration sensors.

## Figures and Tables

**Table 1:** Loading test conditions for the PRSM.

Number	Name	Value	Unit
1	load	30	kN
2	rate of loading	5	kN/s
3	running distance	50 mm	mm
4	velocity	1	mm/s
5	number of cycles per set	3	
6	torque acquisition frequency	50	Hz

**Table 2:** Conditions for testing the initial transmission accuracy of the PRSM.

Number	Name	Value	Unit
1	Load	0	kN
2	Testing distance	50	mm
3	Speed	1	mm/s
4	Number of cycles	1	
5	Collection points	25	

**Table 3:** Structural parameters of the PRSM.

Name	Value	Name	Value
The middle diameter of the screw $d_{s}/mm$	19.5	pitch p/mm	0.4
The middle diameter of the roller $d_{r}/mm$	6.5	Thread profile half angle β/(°)	45
The middle diameter of the nut $d_{n}/mm$	32.5	Number of rollers N	11
Number of starts $n_{s}$	5	Number of starts $n_{r}$	1
Number of starts $n_{n}$	5		

**Table 4:** Conditions set for loading test of the PRSM.

Number	Name	Value	Unit
1	Load	30	kN
2	Rate of loading	5	kN/s
3	Movement distance	50	mm
4	Speed	1	mm/s
5	Number of cycles per set	5	
6	Vibration acquisition frequency	20	KHz
7	Torque acquisition frequency	50/20K	Hz
8	Temperature acquisition frequency	50	Hz

**Table 5:** The variance value of the vibration signal.

Number	Vibration Sensor #1	Vibration Sensor #2	Vibration Sensor #3	Vibration Sensor #4
Group 1	0.000817	0.000001	1.669087	0.000635
Group 5	0.000824	0.000001	1.758934	0.000348
Group 10	0.000711	0.000001	1.226801	0.000151
Group 12	3.391489	0.000018	0.836945	0.000275

## Data Availability

Data is unavailable due to privacy or ethical restrictions.
